# CBT-based Online Self-help Training to Reduce Fear and Distress After Cancer (CAREST Randomized Trial): 24 Months Follow-up Using Latent Growth Models and Latent Class Analysis

**DOI:** 10.1093/abm/kaac078

**Published:** 2023-04-20

**Authors:** Sanne Jasperine van Helmondt, Paul Lodder, Rosalie van Woezik, Jolanda de Vries, Marije Liesbeth van der Lee

**Affiliations:** Department of Medical and Clinical Psychology, Tilburg University, PO Box 90153, 5000 LE Tilburg, The Netherlands; Scientific Research Department, Helen Dowling Institute, Professor Bronkhorstlaan 20, 3723 MB Bilthoven, The Netherlands; Department of Medical and Clinical Psychology, Tilburg University, PO Box 90153, 5000 LE Tilburg, The Netherlands; Department of Methodology and Statistics, Tilburg University, PO Box 90153, 5000 LE Tilburg, The Netherlands; Scientific Research Department, Helen Dowling Institute, Professor Bronkhorstlaan 20, 3723 MB Bilthoven, The Netherlands; Department of Medical and Clinical Psychology, Tilburg University, PO Box 90153, 5000 LE Tilburg, The Netherlands; Admiraal de Ruyter Hospital (Adrz), PO Box 15, 4460 AA Goes, The Netherlands; Department of Medical and Clinical Psychology, Tilburg University, PO Box 90153, 5000 LE Tilburg, The Netherlands; Scientific Research Department, Helen Dowling Institute, Professor Bronkhorstlaan 20, 3723 MB Bilthoven, The Netherlands

**Keywords:** Breast cancer, Cognitive Behavioral Therapy, eHealth, Fear of Cancer recurrence, Psychological distress, Trajectories

## Abstract

**Background:**

Psychological distress (PD) and fear of cancer recurrence (FCR) are common consequences of surviving cancer. Online self-help training could help many cancer survivors deal with PD and FCR at low costs.

**Purpose:**

To evaluate the long-term effectiveness of the CAncer REcurrence Self-help Training (CAREST trial) to reduce PD and FCR. Moreover, to evaluate the relation between FCR and PD across time and identify subgroups representing different change trajectories in FCR over time and their predictors.

**Methods:**

This multicenter randomized controlled trial included 262 female breast cancer survivors, assigned to online self-help training or care as usual. Participants completed questionnaires at baseline and four times during the 24-month follow-up. The primary outcomes were PD and FCR (Fear of Cancer Recurrence Inventory). Latent growth curve modeling (LGCM) and repeated measures latent class analysis (RMLCA) were performed, both according to the intention‐to‐treat principle.

**Results:**

LGCM showed no differences between the average latent slope in both groups for both PD and FCR. The correlation between FCR and PD at baseline was moderate for the intervention group and strong for the CAU group and did not significantly decrease over time in both groups. RMLCA revealed five latent classes and several predictors of class membership.

**Conclusions:**

We did not find a long-term effect of the CBT‐based online self-help training in reducing PD or FCR, nor in their relation. Therefore, we recommend adding professional support to online interventions for FCR. Information about FCR classes and predictors may contribute to improvement of FCR interventions.

## Teaser

This study shows that women who survived breast cancer stay distressed and fearful for a long time, and that online self-help training alone is not effective in reducing their fear of cancer recurrence and psychological distress. This study underlines the importance of developing further effective psychological interventions, for example with professional support

## Introduction

Advances in the diagnosis and treatment of (breast) cancer have resulted in increased survival rates. As a result, there is an increasing group of breast cancer survivors that have to cope with the long-term consequences of cancer. Fear of cancer recurrence (FCR) is one of the most common long-term psychological consequences of surviving breast cancer [1, 2]. FCR is defined as “fear, worry, or concern about cancer returning or progressing” [3] and ranges from healthy levels in the majority of breast cancer survivors to clinical levels in 17% of breast cancer [4, 5]. In younger (age 18–45) breast cancer survivors, this prevalence is much higher: 70% experiences clinical levels of FCR [6]. When FCR surpasses the healthy level, it becomes distressing. Psychological distress (PD) is another common consequence of the diagnosis cancer and its treatment [7, 8]. Up to 50% of cancer survivors experience PD, ranging from normal feelings of vulnerability, feeling discouraged, sadness, and fears, to more disabling problems such as anxiety, panic, and depression [7–10]. FCR and PD are associated constructs and FCR is reported to be a precursor of PD [11–13]. Otherwise, previous research also found that a reduction in PD may lead to a significant reduction in FCR [14, 15]. High levels of FCR are associated with higher frequency of breast self-examination [6] and diminished quality of life [5, 11]. Also, both serious PD and FCR are associated with non-adherence to cancer treatment or—screening [6, 16, 17] and higher healthcare use and costs [18–20].

Given the burden of FCR and PD for cancer survivors, there is a need to develop (tailored) interventions to efficiently address FCR and also to reduce PD [11, 21]. Online interventions are promising, because they are easily accessible, have low costs, offer convenience and greater privacy, and patients can work at their own pace [22, 23]. Therefore, we developed “Less fear after cancer”, an online tailored self-help training on the basis of evidence‐based cognitive behavioral therapy (CBT) [24]. This online self-help training was primarily developed to reduce FCR, but we also expected it to reduce PD because most of its components (e.g., the basic principles of CBT, behavioral techniques to stop ruminating, or relaxation practices) are more widely applicable than FCR alone [24]. This intervention was evaluated within the CAncer REcurrence Self-help Training (CAREST) randomized controlled trial (RCT), in which the intervention was compared to a care as usual (CAU) control group in women with curatively treated breast cancer (breast cancer survivors), with a follow-up period of 24 months [24, 25]. The evaluation of short-term effects at 3 and 9 months showed no treatment effects of the self-help training for FCR [25].

In the current paper, we will evaluate the long-term effectiveness (>15 months) of this online self-help training in reducing both FCR and PD and identify subgroups representing different change trajectories in FCR over time and their predictors. To meet this aim, we use three longitudinal latent variable models, each focusing on a different research question. First, in a multi-group latent growth model, we will evaluate change in FCR and PD (research question 1; RQ1). We hypothesize greater FCR and PD reduction in the intervention group compared to CAU at 15 and 24 months. Second, in a multivariate multi-group latent growth model, we will evaluate the relation between FCR and PD at baseline and across time (research question 2; RQ2). We hypothesized a strong relation (*r* ≥ .50) between FCR and PD at baseline and a significantly weaker relation after the intervention, because PD is expected to decrease more than FCR. In other words, we expected that patients who received the intervention perceive FCR less of a burden, because they have learned to cope with it better. Third, we will use repeated measures latent class analysis (RMLCA) to identify subgroups (“latent classes”) representing different change trajectories in FCR over time (research question 3; RQ3). In our protocol paper, we hypothesized that this intervention would not help all participants, but that there are subgroups to be found for whom this would be beneficial [24]. In the current study, we will investigate the predictors of subgroup membership to identify the characteristics of survivors that benefited most from the online self-help intervention. We will investigate the following potential predictors: socio-demographic and medical variables (age, living alone, rehabilitation, psychological care, chemotherapy, and education), self-efficacy for online self-help (general internet use, health-related behavior, and expectations for online self-help), and psychosocial problems and risk factors (physical problems, depressive symptoms, trait anxiety, and social support).

## Materials and Methods

The CAREST trial was registered in the Dutch Trial Register (NTR4119). The Medical Ethical Committee of the Maasstad hospital (TWOR) in Rotterdam approved this trial (reference number 2013/41) and all participating hospitals provided local ethics approval. The study protocol of this trial has been published [24]. Furthermore, this method section is largely published before in an earlier publication about the short-term treatment effects of this RCT, measures and statistical analyses are adapted for the current article [25]. We conducted this study in accordance with the principles of the Declaration of Helsinki [26], and we reported this study in accordance to the CONSORT guidelines [27].

### Design

We conducted a multicenter randomized controlled trial (RCT) with two conditions. The intervention group received a CBT-based online self-help training to reduce FCR. Both the intervention group and the control group received care as usual (CAU), standard care in their own hospital.

The original sample size calculation mentioned in the study protocol does not apply for the statistical analyses in the current study [24]. In general, for growth models a sample size of at least 100 is preferred [28]. For this study, an alpha of .05 and *df* of 100 (in our model, *df* = 123) results in an estimated power between .87 (for a sample size of 200) and .99 (for a sample size of 300), to reject the null hypothesis of RMSEA ≤ .05 if the true model only moderately fits the data (i.e., RMSEA = .08) [29]. Therefore, the current sample of 262 is fit to perform Covariance Structure Modeling.

Participants were assessed through online self-report questionnaires at baseline (T0), three months (T1; after intervention), nine months (T2), 15 months (T3), and 24 months (T4). More details on the study design are described in the study protocol [24].

### Participants

Women were eligible to participate when they had a diagnosis of breast cancer 1–5 years ago, had no signs of local or regional recurrence or metastatic disease (according to their oncologist or oncology nurse), were capable of completing questionnaires in Dutch, were 18 years or older at disease onset, and had access to a computer with an internet connection [24, 25].

### Recruitment

Eligible patients were recruited consecutively during their regular checkup at the outpatient clinic of a hospital or by sending them a comprehensive information letter by mail (differed because of pragmatic reasons) between April 2014 and May 2016. Participating hospitals (all situated in the Netherlands) were the Maasstad hospital in Rotterdam, St Antonius hospital in Utrecht, Admiraal de Ruyter hospital in Vlissingen, Reinier de Graaf hospital in Delft, Antonius hospital in Sneek, ETZ in Tilburg, Catharina hospital in Eindhoven, and UMCG in Groningen. After 2 weeks, patients were reminded of the study by the researcher. Patients who decided to participate, returned a signed informed consent form. On this form, they indicated whether they agreed to participate in the RCT or only to complete the Dutch version of the Fear of Cancer Recurrence Inventory (FCRI-NL) once [25].

### Randomization

Participants were randomly allocated to either the online self-help training (intervention) or CAU with an allocation ratio of 1:1, stratified by hospital. Block randomization (block size 10) was carried out through the sealed envelope system. Randomization was conducted by a researcher who was not otherwise involved in the study. The researchers had no influence on (and were blinded for) the randomization process. We used coding and separating personal data from the research data to anonymize the data [24, 25].

### Intervention

In short, the intervention is a CBT-based online self-help to reduce FCR. The program included two generic modules containing psycho-education about FCR and the basic principles of CBT, and four optional modules: (a) Stop worrying; (b) Positive actions; (c) Relax; and (d) Reassurance [25]. More information about the intervention can be found in Electronic Supplementary Material 1. All participants received CAU.

### Measures

Socio-demographic and medical variables were self-reported and assessed at baseline and at all time points, respectively. The self-report questionnaires were assessed at all time points.

The primary outcomes FCR and PD were both assessed with the Dutch translation of the Fear of Cancer Recurrence Inventory (FCRI-NL) [30, 31]. The FCRI-NL consists of 42 items on a five-point Likert scale and comprises seven subscales [30]. PD was assessed with the PD subscale (4 items; range 0–16) and FCR was assessed with the severity subscale or FCRI-SF-NL (9 items; range 0–36), which can be used to screen for clinical levels of FCR [30–32]. A cutoff score of 13 or higher on the severity subscale is considered optimal for detecting the presence of clinically significant FCR [32]. Both subscales demonstrated sufficient reliability and validity [31].

Since there was no measure for self-efficacy for online self-help available, self-efficacy was assessed with a 15-item questionnaire developed for this RCT and described in the study protocol, which measured: (1) general internet use (3 items); (2) health-related behavior (7 items); and (3) patients’ expectations of online self-help training for FCR (5 items) [24, 25]. In this patient sample, the estimated reliability of this questionnaire was good (Cronbach’s *α* = .82 for the total scale and Cronbach’s *α* = .72–.93 for the subscales) [25].

Psychosocial problems and risk factors were assessed with the Psychosocial Distress Questionnaire-Breast Cancer (PDQ-BC), a 35-item multidimensional screening instrument specific for breast cancer patients [33]. We used the subscales measuring trait anxiety (10 items), (lack of) social support (1 item), depressive symptoms (7 items), and physical problems (4 items) in this study [25]. The PDQ-BC showed good estimated reliability (Cronbach’s *α* = .70–.87) for the subscales used in this study, and satisfactory construct validity for the physical problems subscale (other subscales were not reported) [25, 33–35].

### Statistical Analyses

Prior to data analysis, we checked the normality assumptions. When the assumption of multivariate normality was not met, we executed transformations of the dependent variable. When transformation did not lead to multivariate normality, we used robust maximum likelihood estimation (MLR) to fit the model. *p*-values smaller than .05 were considered statistically significant. Data were analyzed according to the intention-to-treat (ITT) principle, as mentioned in the study protocol [24]. Missing data were analyzed for random occurrence with Little’s MCAR test for missing data [36]. Multiple imputations were used for all variables to handle the missing data on item level when data was missing completely at random [37,38]. We handled missing data due to attrition using the full information maximum likelihood procedures incorporated in the growth model function of the Lavaan R package. Differences in number of dropouts between both groups were compared with a cross table and chi-square test.

### RQ1: Long-Term Effectiveness of Online Self-Help Training in Reducing FCR and PD

To answer the first research question (i.e., do the intervention and CAU group differ in the change in FCR/PD over time), a multi-group second-order latent growth model was estimated. A second-order growth model estimates individual growth curves for each participant based on the individual FCR or PD item scores. A multi-group extension allows testing differences between interventions in the growth model parameters. The parameter of main interest was the average latent slope estimate in both the intervention and CAU groups and whether these differed between the groups. We estimated the model separately for FCR and PD, because the multivariate model for both FCR and PD did not converge (most likely, the model was too large for the data). Each of the FCR/PD models was built in five steps, first testing the assumption of longitudinal measurement invariance, followed by two models to test the null hypothesis that the difference between the groups in the latent slope is equal to zero. Growth model analyses were estimated with maximum likelihood estimation, using R statistical software (version 3.5.3), with software packages Lavaan (R package for Structural Equation Modeling, version 0.6-9) and BaylorEdPsych (R-package used for checking missing data for random occurrences, version 0.5) [36, 39–41]. The code used for the analyses in this study has been made openly available in the Open Science Framework (OSF) repository (https://osf.io/2sg6m/).

### RQ2: Relation Between FCR and PD Over Time

Answering the second research question (i.e., does the association between FCR and PD decrease over time), we started with a multivariate multi-group growth model including both FCR and PD scores over time. However, as this multivariate second order growth model did not converge, we used a first order growth model that models the growth parameters based on FCR and PD factor scores at each time point instead of on the questionnaire item scores. We first estimated the factor scores of the FCR and PD measurement models at each of the five time points, and subsequently used these factor scores in a multivariate multi-group first order growth model (Model 5) to investigate how change in FCR scores over time was related to change in PD scores over time and how these relations differed between the CAU and intervention group. To test whether the correlations between FCR and PD were equal over time, we added restrictions in Models 6 and 7, respectively, to the intervention and CAU group. We assessed and interpreted the goodness-of-fit of the latent growth models as described above.

### RQ3: Change Trajectories in FCR Over Time and Their Predictors

For the last research question (i.e., can we distinguish characteristics of patients for whom the intervention worked), we modeled a repeated measures latent class analysis (RMLCA) within the intervention group. RMLCA analyses were performed with Latent Gold (version 5.0.0) [42]. The first step of this analysis was to identify the optimal number of latent classes using the BIC, AIC, and AIC3 fit indices, where each class represents a different pattern of change in FCR over time. When not all indices indicated the same number of latent classes, the model supported by most indices was chosen. The second step of the RMCLA was to investigate which predictor variables (as mentioned in the introduction) predict class membership, using Latent Gold’s omnibus Wald test of differences between the latent classes on the predictor variables. Since we assessed multiple predictors, we adjusted the significance level using the Bonferroni–Holm correction [43]. For significant predictors, we used *Z* values >2 or <−2 to determine which class(es) significantly contributed to the predictor effects. Positive/negative values show that participants in that class show higher/lower than average scores on that predictor.

Electronic Supplementary Material 2 contains an extended and more detailed version of this paragraph.

## Results

### Response Rate and Sample Characteristics

**Fig. 1.**
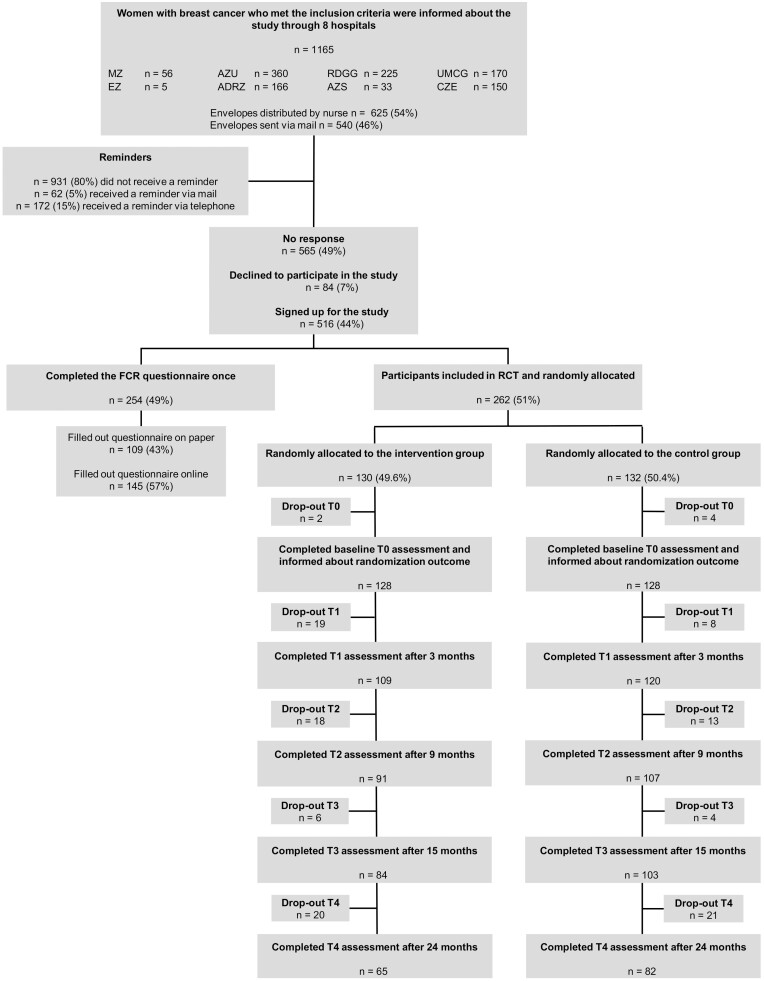
CONSORT diagram for participant inclusion and retention up to 24 months in the CAREST randomized controlled trial. *MZ* Maasstad hospital Rotterdam; *EZ* Elisabeth hospital Tilburg; *AZU* St Antonius Hospital Utrecht; *ADRZ* Admiraal de Ruyter Hospital Vlissingen; *RDGG* Reinier de Graaf Hospital Delft; *AZS* Antonius Hospital Sneek; *UMCG* The University Medical Center Groningen; *CZ* Catharina Hospital Eindhoven; *control group* care as usual (CAU); *intervention group* online self-help for fear of cancer recurrence; *RCT* randomized controlled trial; *T0* baseline measure, before intervention; *T1* 3 months after inclusion, directly after intervention; *T2* 9 months after inclusion; *T3* 15 months after inclusion; *T4* 24 months after inclusion.

Details about enrollment, intervention allocation, follow-up, and drop-out can be found in the flowchart (Fig. 1). In total, 516 (44%) patients gave their consent to participate, of which 254 (49%) chose to complete the FCRI only once (nonparticipants) and 262 (51%) signed up for the RCT (participants). Reasons for drop‐out were personal circumstances (16), not experiencing FCR (8), questionnaires were too burdensome (14), technical difficulties (5), intervention (1), randomization outcome (2), cancer recurrence and/or deceased (3), or unknown (66). Participants in the two RCT conditions did not differ from one another in number of dropouts (*χ*^2^ = 8.33, *p* = .14).

Women in the RCT had a mean age of 55.8 years (*SD* 9.9) and the majority was medium (48%) to highly (40%) educated. Data on participants’ race/ethnicity were not collected. The majority (86%) had a partner and a small number of women (12%) had children younger than 12-years old. Time since diagnosis was on average 2.6 years (SD 1.1) and 40% had affected lymph nodes. Most reported treatments for breast cancer were radiotherapy (68%), chemotherapy (63%), lumpectomy (57%), hormone therapy (56%), and mastectomy (47%). One third of the women reported to have followed a previous rehabilitation program (36%) or previous psychological care (32%). Baseline FCR was 15.2 (SD 6.9). More details about the response rate and the demographic and medical characteristics of the participants were described in an earlier publication [25].

### RQ1: Long-Term Effectiveness of Online Self-help Training in Reducing FCR and PD

When testing the assumption of longitudinal measurement invariance for FCR, the multi-group univariate second order latent growth model indicated no differences between the loadings of the five longitudinal factors across time, based on the chi-square difference test comparing sub-models 0a and 1a (Table 1). Thus, constraining the loadings to be equal across time did not result in a significantly worse model fit [*χ*^2^(63) = 59.38, *p* =.61]. Furthermore, the intercepts of individual items differed significantly across time, based on the chi-square difference test comparing sub-models 1a and 2a (Table 1). Because constraining the intercepts of individual items across time did result in a significantly worse model fit [*χ*^2^(80) = 111.87, *p* < .05], we assumed invariant loadings across time, but we allowed the item intercepts to vary. To find out whether the intervention arms differ in their change in FCR across time, the chi-square difference test comparing sub-models 3a and 4a indicates that the average latent FCR slopes remain equal across groups [*χ*^2^(1) = 0.00, *p* = 1]. This means that FCR did not reduce more in the intervention group than in CAU across time.

**Table 1:** Latent Growth Models Supporting Research Questions 1 and 2, Increasing in Complexity

Model		Parameters (df)	*χ* ^2^ ^a^	*χ* ^2^ difference	AIC^b^	BIC^b^	RMSEA^c^ (95% CI)	CFI^d^	TLI^d^
RQ1a: Multi-group univariate second order latent growth model for fear of cancer recurrence (FCR)									
0a	Baseline model with five correlated longitudinal latent factors (one at each time-point)	500 (1660)	2520.7	–	20098	21871	.064 (.059 to .069)	.882	.859
1a	Model 0a + the loadings of the five longitudinal factors were constrained to be equal across time	437 (1723)	2580.1	59.38	20031	21581	.062 (.057 to .067)	.883	.865
2a	Model 1a + the intercepts of individual items were constrained to be equal across time	357 (1803)	2692.0	111.87^*^	19983	21249	.062 (.057 to .067)	.878	.866
3a	Model 1a + the latent growth factors (intercept and slope)	426 (1734)	2602.7	–	20032	21542	.063 (.058 to .067)	.881	.864
4a	Model 3a + the mean latent slope of the intervention and CAU groups was constrained to be equal	425 (1735)	2602.7	0.00	20030	21537	.063 (.058 to .067)	.881	.864
RQ1b: Multi-group univariate second order latent growth model for psychological distress (PD)									
0b	Baseline model with five correlated longitudinal latent factors (one at each time-point)	238 (222)	345.78	–	9172.1	10015.8	.066 (.052 to .079)	.962	.934
1b	Model 0b + the loadings of the five longitudinal factors were constrained to be equal across time	204 (256)	368.53	22.75	9126.8	9850.0	.059 (.045 to .072)	.965	.948
2b	Model 1b + the intercepts of individual items were constrained to be equal across time	168 (292)	434.16	65.64^**^	9120.5	9716.1	.062 (.049 to .074)	.956	.943
3b	Model 1b + the latent growth factors (intercept and slope)	192 (268)	384.72	–	9119.0	9799.7	.058 (.045 to .071)	.964	.949
4b	Model 3b + the mean latent slope of the intervention and CAU groups was constrained to be equal	191 (269)	384.72	0.00	9117.0	9794.1	.058 (.044 to .071)	.964	.949
RQ2: First order growth model based on factor scores									
5	First order growth model	58 (72)	82.973	–	2338.9	2511.9	.046 (.000 to .085)	.992	.990
6	Model 5 + correlation between PD and FCR was constrained to be equal across time in the *intervention* group	54 (76)	84.941	1.97	2332.8	2494.0	.040 (.000 to .080)	.993	.992
7	Model 5 + correlation between PD and FCR was constrained to be equal across time in the *CAU* group	50 (80)	90.964	6.02	2330.9	2480.0	.043 (.000 to .081)	.992	.991

*df* degrees of freedom; *χ*^*2*^ Chi square; *AIC* Akaike Information Criterion; *BIC* Bayesian Information Criterion; *RMSEA* root mean squared error of approximation; *CI* confidence interval; *CFI* comparative fit index; *TLI* Tucker-Lewis index; *RQ* research question; *CAU* care as usual.

^a^Lower *χ*^*2*^ (Chi square) values indicate a better model.

^b^Lower AIC and BIC values indicate a better model.

^c^RMSEA ≤ .06 indicates good model fit.

^d^CFI and TLI ≥ .95 indicates good model fit.

^*^ *p* < .05;

^**^ *p* < .01.

For PD, the results were similar (Table 1). Constraining the loadings to be equal across time did not result in significantly worse model fit [*χ*^2^(34) = 22.75, *p* =.93]. Yet, constraining the intercepts of individual items across time did result in significantly worse model fit [*χ*^2^(36) = 65.64, *p* < .01], so we assumed invariant loadings and varying item intercepts across time. To find out whether the interventions differ in their change in PD across time, the chi-square difference test comparing submodels 3b and 4b indicates that the average latent PD slopes remain equal across groups [*χ*^2^(1) = 0.00, *p* = 1], which means that PD did not reduce more in the intervention group than in the CAU group across time.

For FCR, the RMSEA fit index was slightly higher than the recommended criterion of .06, and for PD the RMSEA fit index was around .06, suggesting an almost adequate fit of these models to the data. The CFI and TLI suggested a somewhat poor fit of the FCR models to the data. The TLI suggested an almost adequate fit and the CFI indicated a good fit for the PD models. Both AIC and BIC suggest a better fit for the models including equal intercepts of individual items across time (2a and 2b) and for the models including equal latent slopes in the intervention and CAU group (4a and 4b). Answering the first research question (difference between the groups in effect of FCR and PD over time), this corroborates the findings mentioned above that long-term effects did not differ between the intervention group and the CAU group over time, for both FCR and PD models. In other words: the self-help was not effective in reducing FCR and PD in the intervention group long-term.

### RQ2: Relation Between FCR and PD Over Time

In line with the findings of the second order growth model, the results of the first order growth model indicate no significant change in FCR across time, for both the intervention (*β* = .041, *p* = .06) and CAU group (*β* = −.000, *p* = .98]. Similarly, no significant change in PD across time was found, for both the intervention [*β* = .028, *p* = .13] and CAU group [*β* = .003, *p* = .83]. At baseline, we found significant positive correlations between FCR and PD; a moderate correlation in the intervention group (*r* = .471) and a strong correlation in the CAU group (*r* = .708). The first order growth model indicated no change in the correlation between PD and FCR across time for both the intervention and CAU group, based on the chi-square difference test comparing sub-models 5 and 6 and 6 and 7 (Table 1). Thus, constraining the correlation between PD and FCR to be equal at each time point did not result in significantly worse model fit in the intervention [*χ*^2^(4) = 1.97, *p* = .74] and CAU group [*χ*^2^(4) = 6.02, *p* = .20].

**Fig. 2.**
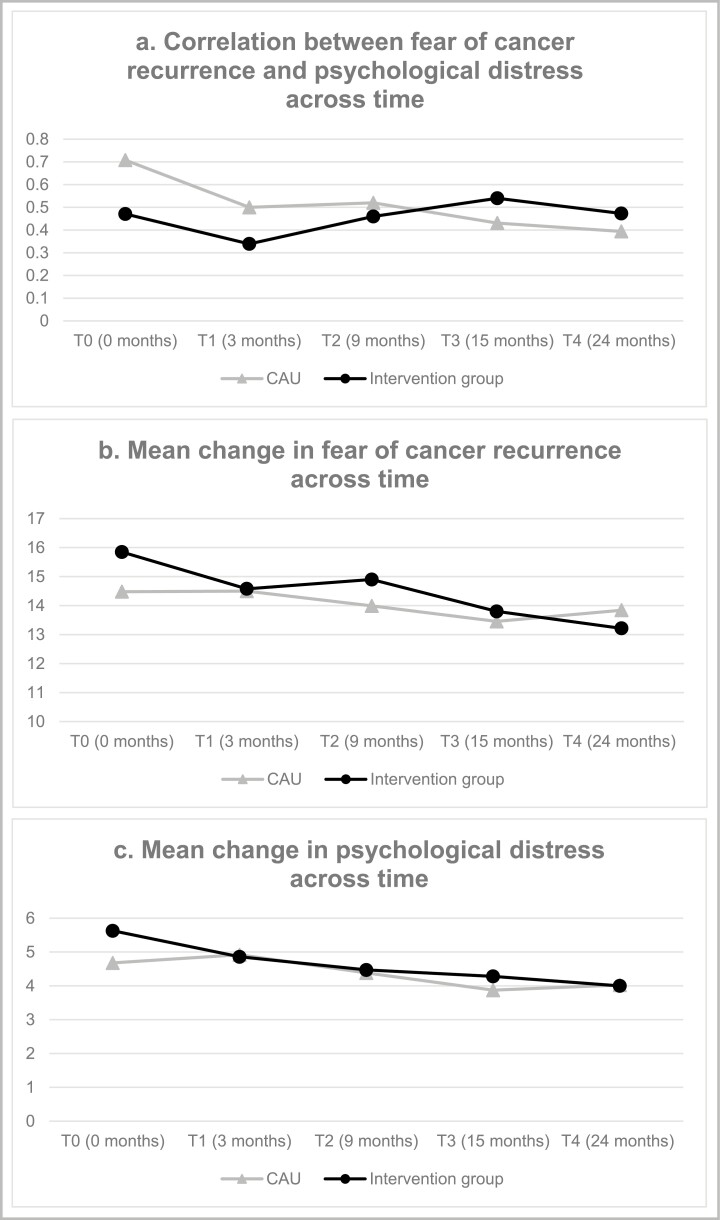
The correlation between fear of cancer recurrence and psychological distress (a), the mean change in fear of cancer recurrence (b) and psychological distress (c) across time for both the intervention and the care as usual (CAU) group.

The results of these chi-square difference tests were confirmed by the model fit indices. Both AIC and BIC suggest a slightly better fit to the data for models 6 and 7 that constrained the correlations between FCR and PD to be equal at each time point. The CFI, TLI and RMSEA similarly indicated good fit for both models 6 and 7 to the data. In short, the model fit indices indicate that the relation between FCR and PD remains equal in both groups over time. Figure 2 illustrates (a) the correlation between FCR and PD, and the mean change in (b) FCR and (c) PD across time for both the intervention and the CAU group. Results show that the correlation between FCR and PD was moderate for the intervention group and strong for the CAU group at baseline and the correlation did not significantly decrease over time in both groups.

### RQ3: Change Trajectories in FCR Over Time and Their Predictors

**Fig. 3.**
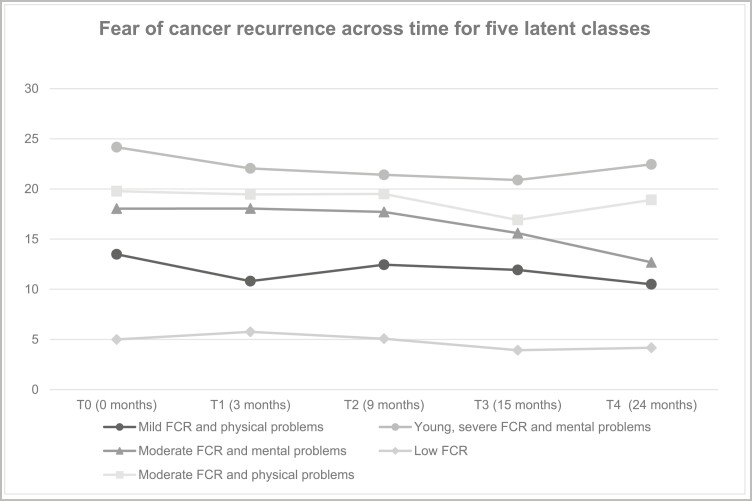
Fear of cancer recurrence (measured with the Fear of Cancer Recurrence Inventory-severity subscale; FCRI-S) across time for five latent classes.

First, we identified the optimal number of latent classes, where each class represents a distinct change pattern of FCRI for participants in the intervention group. BIC showed that a model with three different classes fitted the data best, while AIC and AIC3 suggested a model with five different classes. Because of the consensus between AIC and AIC3, we chose the model with five different classes. For each of those five classes, Fig. 3 shows the mean FCR scores over time.

**Fig. 4.**
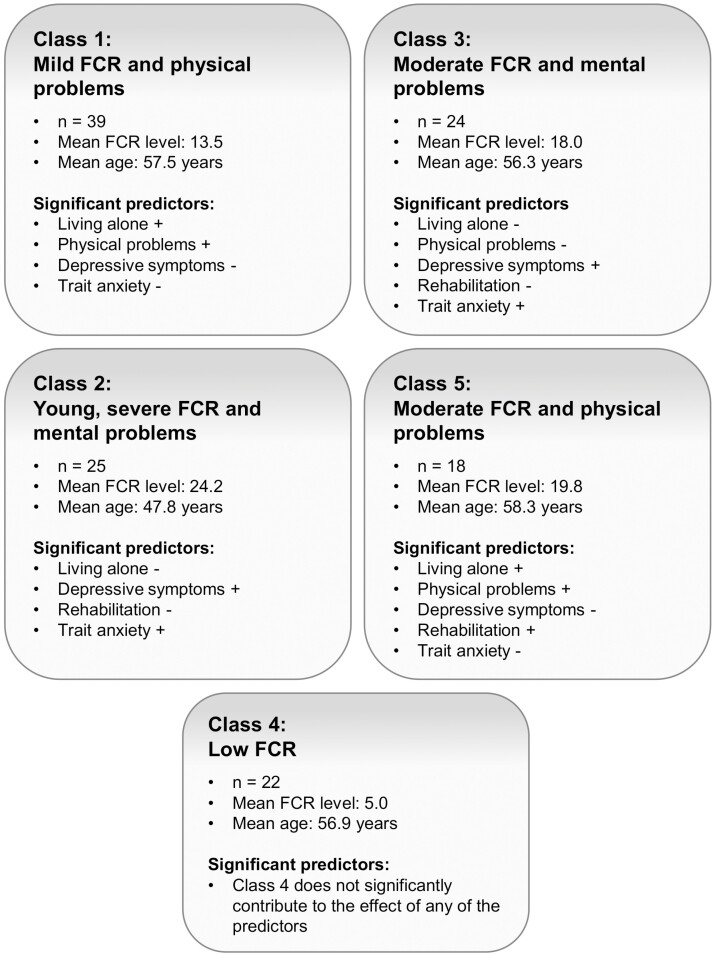
Significant predictors for the latent classes. − Significantly less; + significantly more. *FCR* fear of cancer recurrence.

Second, we determined predictors of class membership. Table 2 shows the effect of various socio-demographic and clinical predictors on class membership. The omnibus test of differences showed that the latent classes differed significantly from each other on the following five predictors: living alone, physical problems, depressive symptoms, having followed rehabilitation, and trait anxiety. Figure 4 gives an overview of the characteristics of the members of the five latent classes based on the results in Table 2. Below, the classes will be described in the same order as Table 2 (largest to smallest class). Class 1 will be named “mild FCR and physical problems class”, because participants in this class have mild FCR (mean FCR severity of 13.5) and predominantly physical symptoms. Class 2 will be named “young, severe FCR and mental problems class”, because participants in this class have severe FCR (mean FCR severity of 24.2), predominantly mental symptoms and a notable lower mean age than all other classes (47.8 years vs. 56.3–58.3 years). Class 3 will be named “moderate FCR and mental problems class”, because participants in this class have moderate FCR (mean FCR severity of 18.0) and predominantly mental symptoms. Class 4 will be named “low FCR class”, because participants in this class have a very low FCR level (mean FCR severity of 5.0) and this class did not significantly contribute to the effect of any of the predictors identified through the omnibus tests. Class 5 will be named “moderate FCR and physical problems class”, because participants in this class have moderate FCR (mean FCR severity of 19.8) and predominantly physical symptoms.

**Table 2:** Clinical and Socio-demographic Predictors of Fear of Cancer Recurrence Latent Class Membership (Research Question 3)

	Class 1	Class 2	Class 3	Class 4	Class 5	Wald test	*p*-value	Bonferroni-Holm adjusted significance level
	*n =* 39	*n =* 25	*n =* 24	*n =* 22	*n =* 18			
Living alone	**2.7677**	**−2.521**	**−3.697**	0.8813	**3.4848**	20.7346^*^	.00036	.00357
Physical problems (PDQ_PH)	**2.1964**	−1.189	**−3.695**	−1.892	**3.8359**	19.8868^*^	.00053	.00385
Depressive symptoms (PDQ_DE)	**−3.13**	**3.5567**	**3.7437**	−1.693	**−2.174**	18.9^*^	.00082	.00417
Rehabilitation	0.8723	**−2.234**	**−3.394**	−0.587	**3.4923**	17.515^*^	.0015	.00455
Trait anxiety (PDQ_AT)	**−2.14**	**2.5195**	**2.8991**	1.6154	**−3.528**	16.6462^*^	.0023	.005
Psychological care	−0.537	2.5382	−3.108	−1.293	2.0092	13.0318	.011	.00556
Expectations online self-help	−1.639	1.3169	−0.616	−3.017	1.8949	10.1127	.039	.00625
General internet use	−2.728	0.4759	1.2917	1.2778	−0.868	8.1693	.086	.00714
Social support (PDQ_SP)	−1.334	0.1144	0.5255	2.4202	−1.932	7.6463	.11	.00833
Children living at home	−1.66	−0.592	2.1218	1.2226	−1.344	7.0531	.13	.01
Health-related behavior	2.4824	−0.769	−1.474	−1.022	0.9706	7.157	.13	.0125
Age	−0.323	−2.42	−0.067	−0.057	1.8432	6.9047	.14	.01667
Chemotherapy	−0.567	2.3266	0.0262	−0.29	−1.521	5.739	.22	.025
Education	−0.133	0.1371	−0.061	−0.184	0.0775	27.7102	.69	.05

Bold values: values of >2 or <−2 are used to determine which class(es) significantly contribute to the effect of the predictor. Positive values show that participants in that class show higher than average scores on that predictor; negative values show that participants in that class show lower than average scores on that predictor.

^*^Significant Wald test: *p*-value smaller than the Bonferroni-Holm adjusted significance level.

Our results show quite stable FCR trajectories for four (out of five) latent classes over time (indicating stable severe, moderate, mild, and low FCR over time). At first view, one of the classes shows a decrease of five points on the severity subscale of the FCRI over 24 months. Looking closer, we see the decrease occurs after 9 months (6 months after the intervention); hence we do not expect this to be a treatment effect of our online self-help. In conclusion, RMLCA did not reveal classes who benefit from the intervention.

## Sensitivity Analyses

More than one-third (37%) of the intervention group reported they did not actually follow the online self-help training, although they had access to the intervention. However, participants who followed the online self-help intervention and participants who did not follow the intervention did not differ from one another in class membership (*χ*^2^ = 2.26, *p* = .69). We have repeated our analysis of RQ1 excluding those participants who had indicated that they did not participate in the online self-help training. For FCR, the results of the sensitivity analyses show a latent slope of −0.09 in the intervention group and −0.05 in the control group, the difference in latent slopes of 0.04 was not statistically significant (*p* = .175). For PD, the results of the sensitivity analyses show a latent slope of −0.11 in the intervention group and −0.05 in the control group, the difference of 0.06 was almost statistically significant (*p* = .052). Thus, sensitivity analyses showed no significant treatment effect for the intervention group compared to CAU.

## Discussion

The current study is one of the few studies that investigate the effect of an online self-help training for FCR with a follow-up of 24 months. Results indicate no change of both FCR and PD across time, for both the intervention and CAU group, nor any effect of the intervention on the relation between FCR and PD, nor did we find a class of participants that benefitted from our online self-help training. Although some *p*-values testing whether a condition showed change over time were almost statistically significant, the expected change across time is very small and not clinically meaningful.

This result is largely in line with a recent literature review about online interventions aimed at reducing FCR and PD in cancer patients. They found partial support for reduction in PD, and limited evidence for reducing anxiety after online interventions [44]. The PD reductions were achieved by interventions in which patients were screened on PD before study inclusion, and interventions which included therapist support [44]. Since other studies showed that interventions with therapist support, standard e-mail reminders, or only including patients with high FCR were more effective, this may explain the differences in results with our study [45–48]. One of the papers from the literature review did not find an intervention-effect in the primary analysis, but secondary per-protocol analysis restricted to patients who accessed at least half of the intervention resulted in a greater decrease in psychological and cancer specific distress compared to a control group [49]. In the current study, secondary analysis did not show an intervention effect for participants who followed the online self-help training, compared to CAU. In line with our study, another systematic review about self-guided interventions for managing PD in people with cancer found no effects in reducing PD in four online self-guided interventions [50]. With the current study, we consciously chose to evaluate the long-term effectiveness of our online self-help training in a realistic setting (how it was intended to use); without screening for high levels of FCR or PD, sending reminders, or therapist support. Such a purely online self-help training has several advantages, such as easy accessibility, low costs, greater privacy, and patients can work at their own pace [22, 23]. In conclusion, despite these advantages, online self-help interventions (without screening or support) may show limited effects. Moreover, in line with previous research [1, 51, 52], our results showed no significant natural decrease of both FCR and PD over time (in both groups), which may indicate that FCR and PD are stable over time.

The baseline correlations between FCR and PD that we found in this study, were in line with previous research [15, 53]. The difference between both groups on baseline correlation between FCR and PD is remarkable, since the groups were randomized. Presumably, this may be an incidental finding. A possible explanation for not finding a decrease in relation between FCR and PD could be the large variability in individual FCR and PD baseline levels.

Moreover, we identified five latent classes that capture different patterns in FCR across time. Our results showed quite stable FCR trajectories for four of the latent classes over time (stable severe, moderate, mild, and low FCR). One of the classes has moderate baseline FCR and starts to decrease 6 months after the intervention, but we do not expect this to be an effect of our online self-help training. Recently, some studies have been published about FCR classes and trajectories [2, 54–61]. However, comparison of these studies is hampered by the use of different study designs, for example duration of the studies, outcome measures, and statistical methods. Therefore, the results of these studies about classes and trajectories in FCR are too disparate to compare them with our results. Likewise, five studies have reported predictors for FCR trajectory classes, but there is so much variability in study design [55, 57, 58, 61, 62], that we cannot compare results.

### Limitations and Strengths

This study had several limitations. First, since R package Lavaan gave a warning for all growth models using both MLR estimation and other robust estimators, ML estimation was used to fit the model. Presumably, the model with several hundred parameters was too large for our data. The ML estimator is not robust against deviations from normally distributed data, which can lead to biased model parameters. This should be considered when interpreting significant results. However, (full information) ML is a good estimator to handle missing data and to use as much data as possible in longitudinal analyses. Second, the high percentage of nonusers may have distorted the results, although this shows probably a realistic picture of usage of online self-help interventions. Sensitivity analyses showed that the nonusers did probably not influence the treatment effect of the intervention: when leaving the participants who did not actually follow the online self-help training out of the analyses, also no significant treatment effect was found for the intervention group compared to CAU. Third, we used a subscale of the FCRI to measure PD, while most other studies used different questionnaires for PD, such as the Hospital Anxiety and Depression Scale (HADS) [44]. This may complicate comparison of the different studies.

An important strength of our study design is the long follow-up period of 24 months. The effect studies mentioned above, reported follow-up periods ranging from immediately after intervention to 12 months after baseline [44, 46, 47, 49, 50]. From the studies about FCR classes and trajectories in FCR, only two reported follow-up periods above 24 months: 5 years (a prospective cohort of women diagnosed with breast cancer at age ≤40 years) and 9 years (a national prospective longitudinal study from the American Cancer Society’s Study of Cancer Survivors-I) [55, 61]. This illustrates that the follow-up period of 24 months in our RCT is valuable and adds to the body of knowledge of FCR. Second, we used state-of-the-art analysis. When analyzing RCT data consisting of repeated measurements of a psychological construct, a latent growth model outperforms a traditional observed score model because the latter does not take into account the measurement error in the questionnaire item scores and therefore tends to produce biased treatment effects [63]. Also, as found in our earlier publication, the large variation in the change in FCR and PD scores within participants over time in this RCT supports the importance of our choice to model individual differences in growth curves using a latent growth curve models (instead of comparing the average change over time) [25]. Third, as mentioned before, the ecological validity of the current study is high [25]. This study reflects a realistic picture of online self‐help interventions, because we did not screen for high levels of FCR, CAU was allowed, and we offered purely self-help without extra help or emails. Other strengths (also mentioned in an earlier publication) were the use of a large consecutive sample and the fact that our study sample consisted of relatively young women [25]. Since young women use the internet more often than older women for both searching for information and for personal development, and the prevalence of FCR in young breast cancer survivors is high, we did reach the right target group with this RCT [6, 64].

### Clinical Implications

Levels of FCR and PD seem to be stable over time (showed no significant natural decrease), which underlines the importance to develop interventions for both FCR and PD in breast cancer survivors. In the current study, we found no (long-term) effect of our online self-help training. To retain the benefits of online interventions, such as easy accessibility, low costs, and work at their own pace, we suggest to study whether online interventions with professional support, for example, from general practice mental health professionals (GP-MHPs) might be effective (as also suggested in our earlier publication) [25]. This RCT showed five possible FCR subgroups representing different trajectories in FCR over time and their predictors, which is important information that adds to the body of knowledge about FCR. Knowing more about the specific needs of each FCR group may contribute to the development of better interventions. More research is needed to confirm our results.

## Conclusions

In conclusion, five latent classes and several predictors of class membership were found. Knowing more about the specific needs of FCR groups, may contribute to the improvement of interventions for FCR.

Furthermore, there was no (long-term) effect of the CBT‐based online self-help training “Less fear after cancer” in reducing both FCR and PD. Therefore, we recommend adding professional support, like email contact or face‐to‐face assistance, to future online interventions for FCR and PD.

## Supplementary Material

kaac078_suppl_Supplementary_Materials

## References

[1] Koch L , Jansen L, Brenner H, Arndt V. Fear of recurrence and disease progression in long-term (≥ 5 years) cancer survivors—a systematic review of quantitative studies. Psychooncology. 2013; 22(1):1–11. 10.1002/pon.3022 22232030

[2] Heidkamp P , Breidenbach C, Hiltrop K, et al., Individual courses and determinants of fear of cancer recurrence in long-term breast cancer survivors with and without recurrence. Support Care Cancer. 2021; 29(12):7647–7657. 34137933 10.1007/s00520-021-06329-z PMC8549971

[3] Lebel S , Ozakinci G, Humphris G, et al., From normal response to clinical problem: definition and clinical features of fear of cancer recurrence. Support Care Cancer. 2016; 24(8):3265–3268. 27169703 10.1007/s00520-016-3272-5

[4] Simonelli L , Siegel S, Duffy N. Fear of cancer recurrence: a theoretical review and its relevance for clinical presentation and management. Psychooncology. 2017; 26(10):1444–1454. 27246348 10.1002/pon.4168

[5] Koch L , Bertram H, Eberle A, et al., Fear of recurrence in long-term breast cancer survivors - still an issue. Results on prevalence, determinants, and the association with quality of life and depression from the Cancer Survivorship—a multi-regional population-based study. Psychooncology. 2014; 23(5):547–554. 24293081 10.1002/pon.3452

[6] Thewes B , Butow P, Bell ML, et al., Fear of cancer recurrence in young women with a history of early-stage breast cancer: a cross-sectional study of prevalence and association with health behaviours. Support Care Cancer. 2012; 20(11):2651–2659. 22328003 10.1007/s00520-011-1371-x

[7] Riba MB , Donovan KA, Andersen B, et al., Distress management, version 3.2019, NCCN clinical practice guidelines in oncology. J Natl Compr Canc Netw. 2019; 17(10):1229–1249. 31590149 10.6004/jnccn.2019.0048 PMC6907687

[8] Mehnert A , Hartung TJ, Friedrich M, et al., One in two cancer patients is significantly distressed: prevalence and indicators of distress. Psychooncology. 2018; 27(1):75–82. 28568377 10.1002/pon.4464

[9] Chambers SK , Girgis A, Occhipinti S, et al., Psychological distress and unmet supportive care needs in cancer patients and carers who contact cancer helplines. Eur J Cancer Care. 2012; 21(2):213–223. 10.1111/j.1365-2354.2011.01288.x 21895814

[10] Gundelach A , Henry B. Cancer-related psychological distress: a concept analysis. Clin J Oncol Nurs. 2016; 20(6):630–634. 27857256 10.1188/16.CJON.630-634

[11] Simard S , Thewes B, Humphris G, et al., Fear of cancer recurrence in adult cancer survivors: a systematic review of quantitative studies. J Cancer Surviv. 2013; 7(3):300–322. 23475398 10.1007/s11764-013-0272-z

[12] Costanzo ES , Lutgendorf SK, Mattes ML, et al., Adjusting to life after treatment: distress and quality of life following treatment for breast cancer. Br J Cancer. 2007; 97(12):1625–1631. 18000503 10.1038/sj.bjc.6604091 PMC2360272

[13] Lee-Jones C , Humphris G, Dixon R, Hatcher MB. Fear of cancer recurrence—a literature review and proposed cognitive formulation to explain exacerbation of recurrence fears. Psychooncology. 1997; 6(2):95–105. 9205967 10.1002/(SICI)1099-1611(199706)6:2<95::AID-PON250>3.0.CO;2-B

[14] Lebel S , Rosberger Z, Edgar L, Devins GM. Emotional distress impacts fear of the future among breast cancer survivors not the reverse. J Cancer Surviv. 2009; 3(2):117–127. 19322661 10.1007/s11764-009-0082-5

[15] Hodges LJ , Humphris GM. Fear of recurrence and psychological distress in head and neck cancer patients and their carers. Psychooncology. 2009; 18(8):841–848. 19101920 10.1002/pon.1346

[16] Mausbach BT , Schwab RB, Irwin SA. Depression as a predictor of adherence to adjuvant endocrine therapy (AET) in women with breast cancer: a systematic review and meta-analysis. Breast Cancer Res Treat. 2015; 152(2):239–246. 26077640 10.1007/s10549-015-3471-7 PMC4861253

[17] Lin C , Clark R, Tu P, Bosworth HB, Zullig LL. Breast cancer oral anti-cancer medication adherence: a systematic review of psychosocial motivators and barriers. Breast Cancer Res Treat. 2017; 165(2):247–260. 28573448 10.1007/s10549-017-4317-2

[18] Han X , Lin CC, Li C, et al. Association between serious psychological distress and health care use and expenditures by cancer history. Cancer. 2015; 121(21):614–622. 25345778 10.1002/cncr.29102 PMC4492528

[19] Compen FR , Adang EMM, Bisseling EM Van der Lee ML Speckens AEM. Exploring associations between psychiatric disorder, psychological distress, and health care utilization in cancer patients. Psychooncology. 2018; 27(3):871–878. 29205675 10.1002/pon.4591

[20] Lebel S , Tomei C, Feldstain A, Beattie S, McCallum M. Does fear of cancer recurrence predict cancer survivors’ health care use? Support Care Cancer. 2013; 21(3):901–906. 23269420 10.1007/s00520-012-1685-3

[21] Ziner KW , Sledge GW, Bell CJ, Johns S, Miller KD, Champion VL. Predicting fear of breast cancer recurrence and self-efficacy in survivors by age at diagnosis. Oncol Nurs Forum. 2012; 39(3):287–295. 22543387 10.1188/12.ONF.287-295 PMC5018900

[22] Christensen H , Batterham P, Calear A. Online interventions for anxiety disorders. Curr Opin Psychiatry. 2014; 27(1):7–13. 24257123 10.1097/YCO.0000000000000019

[23] Andrews G , Cuijpers P, Craske MG, McEvoy P, Titov N. Computer therapy for the anxiety and depressive disorders is effective, acceptable and practical health care: a meta-analysis. PLoS One. 2010; 5(10):e13196. 20967242 10.1371/journal.pone.0013196 PMC2954140

[24] van Helmondt SJ , van der Lee ML, de Vries J. Study protocol of the CAREST-trial: a randomised controlled trial on the (cost-) effectiveness of a CBT-based online self-help training for fear of cancer recurrence in women with curatively treated breast cancer. BMC Cancer. 2016; 16:527. 27455846 10.1186/s12885-016-2562-0 PMC4960756

[25] van Helmondt SJ , van der Lee ML, van Woezik RAM, Lodder P, de Vries J. No effect of CBT-based online self-help training to reduce fear of cancer recurrence: first results of the CAREST multicenter randomized controlled trial. Psychooncology. 2020; 29(1):86–97. 31595627 10.1002/pon.5233

[26] World Medical Association. World Medical Association Declaration of Helsinki: ethical principles for medical research involving human subjects. JAMA. 2013; 310(20):2191–2194. 24141714 10.1001/jama.2013.281053

[27] Schulz KF , Altman DG, Moher D. CONSORT 2010 statement: updated guidelines for reporting parallel group randomised trials. BMC Med. 2010; 8:18. 20334633 10.1186/1741-7015-8-18 PMC2860339

[28] Curran PJ , Obeidat K, Losardo D. Twelve frequently asked questions about growth curve modeling. J Cogn Dev. 2010; 11(2):121–136. 21743795 10.1080/15248371003699969 PMC3131138

[29] MacCallum RC , Browne MW, Sugawara HM. Power analysis and determination of sample size for covariance structure modeling. Psychol Methods. 1996; 1(2):130–149.

[30] Simard S , Savard J. Fear of Cancer Recurrence Inventory: development and initial validation of a multidimensional measure of fear of cancer recurrence. Support Care Cancer. 2009; 17(3):241–251. 18414902 10.1007/s00520-008-0444-y

[31] van Helmondt SJ , van der Lee ML, de Vries J. Translation and validation of the Dutch version of the Fear of Cancer Recurrence Inventory (FCRI-NL). J Psychosom Res. 2017; 102:21–28. 28992893 10.1016/j.jpsychores.2017.09.001

[32] Simard S , Savard J. Screening and comorbidity of clinical levels of fear of cancer recurrence. J Cancer Surviv. 2015; 9(3):481–491. 25603948 10.1007/s11764-015-0424-4

[33] Bogaarts MPJ , Den Oudsten BL, Roukema JA, Van Riel JMGH, Beerepoot LV, De Vries J. Development of the Psychosocial Distress Questionnaire-Breast Cancer (PDQ-BC): a breast cancer-specific screening instrument for psychosocial problems. Support Care Cancer. 2011; 19(10):1485–1493. 20814700 10.1007/s00520-010-0968-9 PMC3166599

[34] Bogaarts MPJ , Den Oudsten BL, Roukema JA, Van Riel JMGH, Beerepoot LV, De Vries J. The Psychosocial Distress Questionnaire-Breast Cancer (PDQ-BC) is a useful instrument to screen psychosocial problems. Support Care Cancer. 2012; 20(8):1659–1665. 21863369 10.1007/s00520-011-1256-z

[35] Bogaarts MPJ , Den Oudsten BL, Roukema JNA, Van Riel JMGH, Beerepoot LV, De Vries J. Reliability and validity of the psychosocial distress questionnaire-breast cancer. J Nurs Meas. 2014; 22(2):14–28. 26609900 10.1891/1061-3749.22.2.E14

[36] Beaujean AA. BaylorEdPsych: R Package for Baylor University Educational Psychology Quantitative Courses. 2012. Retrieved from https://CRAN.R-project.org/package=BaylorEdPsych.

[37] Little RJA. A test of missing completely at random for multivariate data with missing values. J Am Stat Assoc. 1988; 83:1198–1202.

[38] Resseguier N , Giorgi R, Paoletti X. Sensitivity anaylsis when data are missing not-at-random. Epidemiology. 2011; 22(2):282. 21293212 10.1097/EDE.0b013e318209dec7

[39] IBM Corp. IBM SPSS Statistics for Windows, Version 23.0 Armonk, NY: IBM Corp. 2014.

[40] R Core Team. R: A language and environment for statistical computing. Vienna, Austria: R Foundation for Statistical Computing. 2017. https://www.R-project.org/.

[41] Rosseel Y. lavaan: An R Package for structural equation modeling. J Stat Softw. 2012; 48(2):1–36.

[42] Vermunt JK , Magidson J. Technical Guide for Latent GOLD 5.0: Basic, Advanced, and Syntax. Belmont, MA: Statistical Innovations Inc., 2013.

[43] Holm S. A simple sequentially rejective multiple test procedure. Scand J Stat. 1979; 6(2):65–70.

[44] Willems RA , Bolman CAW, Lechner L, et al. Online interventions aimed at reducing psychological distress in cancer patients: evidence update and suggestions for future directions. Curr Opin Support Palliat Care. 2020; 14(1):27–39. 31895066 10.1097/SPC.0000000000000483

[45] Linden W , Girgis A. Psychological treatment outcomes for cancer patients: what do meta-analyses tell us about distress reduction? Psychooncology. 2012; 21(4):343–350. 21882287 10.1002/pon.2035

[46] Van de Wal M , Thewes B, Gielissen M, Speckens A, Prins J. Efficacy of blended cognitive behavior therapy for high fear of recurrence in breast, prostate, and colorectal cancer survivors: the SWORD Study, a Randomized Controlled Trial. J Clin Oncol. 2017; 35(19):2173–2183. 28471726 10.1200/JCO.2016.70.5301

[47] Van Den Berg SW , Gielissen MFM, Custers JAE, Van Der Graaf WTA, Ottevanger PB, Prins JB. BREATH: Web-based self-management for psychological adjustment after primary breast cancer-results of a multicenter randomized controlled trial. J Clin Oncol. 2015; 33(25):2763–2771. 26169621 10.1200/JCO.2013.54.9386

[48] Spek V , Cuijpers P, Nyklícek I, Riper H, Keyzer J, Pop V. Internet-based cognitive behaviour therapy for symptoms of depression and anxiety: a meta-analysis. Psychol Med. 2007; 37(3):319–328. 17112400 10.1017/S0033291706008944

[49] Chambers SK , Ritterband LM, Thorndike F, et al., Web-delivered cognitive behavioral therapy for distressed cancer patients: Randomized controlled trial. J Med Internet Res. 2018; 20(1):e42. 29386173 10.2196/jmir.8850 PMC5812983

[50] Ugalde A , Haynes K, Boltong A, et al., Self-guided interventions for managing psychological distress in people with cancer—a systematic review. Patient Educ Couns. 2017; 100(5):846–857. 28081937 10.1016/j.pec.2016.12.009

[51] Vandraas KF , Reinertsen KV, Kiserud CE, Lie HC. Fear of cancer recurrence among young adult cancer survivors—exploring long-term contributing factors in a large, population-based cohort. J Cancer Surviv. 2021; 15(4):497–508. 32989672 10.1007/s11764-020-00943-2 PMC8272704

[52] Lebel S , Rosberger Z, Edgar L, Devins GM. Comparison of four common stressors across the breast cancer trajectory. J Psychosom Res. 2007; 63(3):225–232. 17719358 10.1016/j.jpsychores.2007.02.002

[53] Lebel S , Simard S, Harris C, et al., Empirical validation of the English version of the Fear of Cancer Recurrence Inventory. Qual Life Res. 2016; 25(2):311–321. 26341969 10.1007/s11136-015-1088-2

[54] McGinty HL , Small BJ, Laronga C, Jacobsen PB. Predictors and patterns of fear of cancer recurrence in breast cancer survivors. Health Psychol. 2016; 35(1):1–9. 26030308 10.1037/hea0000238

[55] Leclair CS , Lebel S, Westmaas JL. The Relationship between fear of cancer recurrence and health behaviors: A Nationwide Longitudinal Study of Cancer Survivors. Health Psychol. 2019; 38(7):596–605. 31120271 10.1037/hea0000754

[56] Ng DWL , Foo CC, Ng SSM, et al., The role of metacognition and its indirect effect through cognitive attentional syndrome on fear of cancer recurrence trajectories: a longitudinal study. Psychooncology. 2020; 29(2):271–279. 31663187 10.1002/pon.5234

[57] Custers JAE , Kwakkenbos L, van der Graaf WTA, Prins JB, Gielissen MFM, Thewes B. Not as stable as we think: a descriptive study of 12 monthly assessments of fear of cancer recurrence among curatively-treated breast cancer survivors 0–5 years after surgery. Front Psychol. 2020; 11:580979. 33224072 10.3389/fpsyg.2020.580979 PMC7667242

[58] Shim EJ , Jeong D, Lee SB, Min YH. Trajectory of fear of cancer recurrence and beliefs and rates of medication adherence in patients with breast cancer. Psychooncology. 2020; 29(11):1835–1841. 32720375 10.1002/pon.5497

[59] Wu LM , McGinty H, Amidi A, Bovbjerg K, Diefenbach MA. Longitudinal dyadic associations of fear of cancer recurrence and the impact of treatment in prostate cancer patients and their spouses. Acta Oncol. 2019; 58(5):708–714. 30741082 10.1080/0284186X.2018.1563714 PMC6534441

[60] Dunn LB , Langford DJ, Paul SM, et al., Trajectories of fear of recurrence in women with breast cancer. Support Care Cancer. 2015; 23(7):2033–2043. 25524004 10.1007/s00520-014-2513-8 PMC5469210

[61] Schapira L , Zheng Y, Gelber SI, et al., Trajectories of fear of cancer recurrence in young breast cancer survivors. Cancer. 2022; 128(2):335–343. 34614212 10.1002/cncr.33921 PMC9397577

[62] Yang Y , Cameron J, Bedi C, Humphris G. Fear of cancer recurrence trajectory during radiation treatment and follow-up into survivorship of patients with breast cancer. BMC Cancer. 2018; 18(1):1–9. 30342495 10.1186/s12885-018-4908-2 PMC6195993

[63] Gorter R , Fox J, Apeldoorn A, Twisk J. Measurement model choice influenced randomized controlled trial results. J Clin Epidemiol. 2016; 79:140–149. 27394673 10.1016/j.jclinepi.2016.06.011

[64] Van Deursen AJAM , Van Dijk JAGM, Ten Klooster PM. Increasing inequalities in what we do online: a longitudinal cross sectional analysis of Internet activities among the Dutch population (2010 to 2013) over gender, age, education, and income. Telemat Inform. 2015; 32(2):259–272.

