# Supplementary material for: CBT-based Online Self-help Training to Reduce Fear and Distress After Cancer (CAREST Randomized Trial): 24 Months Follow-up Using Latent Growth Models and Latent Class Analysis
Source: Ann Behav Med. 2023 Apr 20;57(9):787–99. doi: 10.1093/abm/kaac078 (PMC10441870; doi:10.1093/abm/kaac078)
Supplement: kaac078_suppl_Supplementary_Materials [file kaac078_suppl_supplementary_materials.docx]

**CBT-based online self-help training to reduce fear and distress after cancer (CAREST randomized trial): 24 months follow-up using latent growth models and latent class analysis**

Electronic Supplementary Material 1

**More information about the online self-help training “Less fear after cancer”**

“Less fear after cancer” is a CBT-based online self-help training to reduce fear of cancer recurrence (FCR), which includes two generic modules about FCR and the basic principles of CBT, and four optional modules. The training is tailored, which means that participants start the training by filling out the Fear of Cancer Recurrence Inventory (FCRI-NL), after which they get (automated) feedback about their scores and a suggestion about which optional modules to follow after the general modules.

The first generic module contains psycho-education about FCR, its symptoms and learning to recognize symptoms of fear, and in the second generic module participants learn about the basic principles of cognitive behavioral therapy (this module is divided in two parts). The four optional modules contain information about making an action plan, behavioral techniques to stop worrying, relaxation practices (with audio files), and information about how and when to seek reassurance.


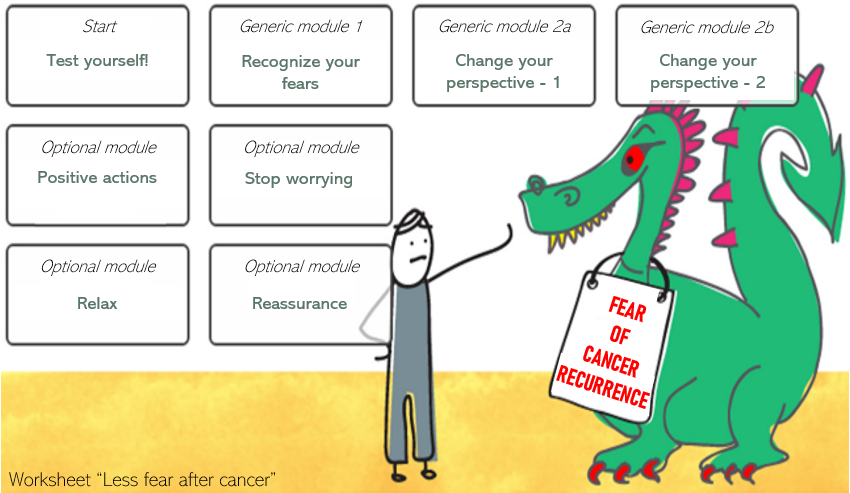
The most important functionality of the training is the worksheet, because it gives an overview of the modules and access to the intervention (translated in English):

Each module consists of an informative part and a practical part:

- The informative part contains more information about the topic, which is presented in both written text and sound clips (participants can choose what they prefer). Also, there are illustrations and videos in which patients talk about their experiences with FCR and the exercises.
- In the practical part, participants are motivated to do exercises or assignments in daily life. By clicking on a module, participants can access all information (texts, videos, audio files) and exercises of that module.

Example of an informative page from generic module 2 (translated in English):

| **Think gray! (and not black-and-white)** |
| --- |
| 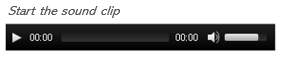  Thoughts are no facts!  Unhelpful thoughts often don’t match reality:   - They are exaggerated. - They are full of prejudices. - They don't draw the right conclusion.   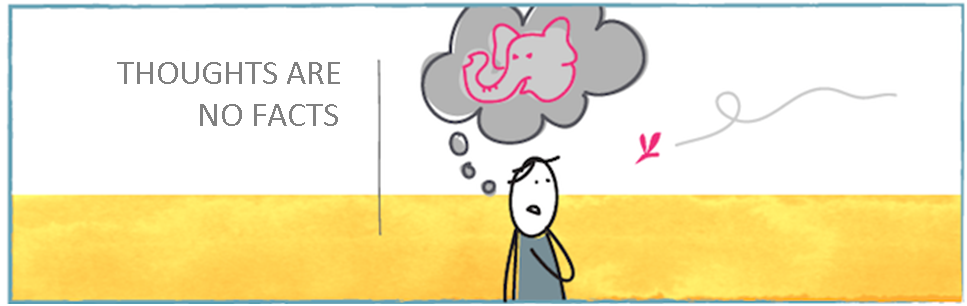 |

Example of an assignment from the optional module “Stop Worrying” (translated in English):

| **Weekly assignment 2: Worry-time** |
| --- |
| 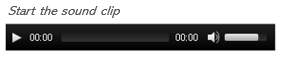  Sometimes worrying can be a relief. But worrying for too long costs a lot of energy! This is at the expense of other activities.  The worry-time technique can help with this.  ***What is worry-time?***   - Set up your worry-time (e.g., 15 minutes). - During this period you can think about your worries. - Set a fixed time of day in which you are allowed to worry. - 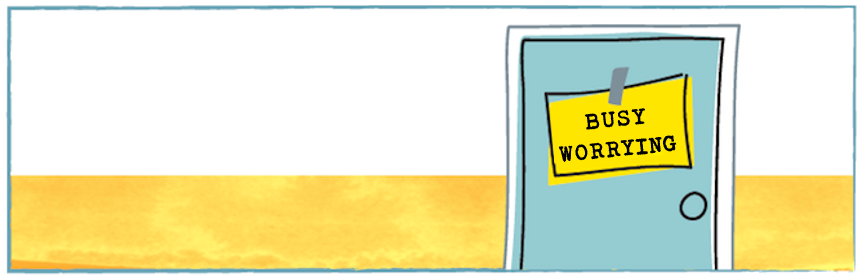Only allow yourself to worry during worry-time. |

We advise participants to spend a week on each module they follow. Also, we explain that the more time they invest, the more effect they can expect from the training. Eventually, participants choose themselves which additional modules they follow and how much time they spend on the training. Most participants will need four to six weeks to complete the training, depending on how many modules they follow. All participants have access to the training for three months.

Other functionalities of the intervention include a library with the information and forms in pdf format, videos, audio files, and a mailbox for technical assistance.

Electronic Supplementary Material 2

**Statistical analyses - Extended and more detailed version**

Prior to data analysis, we checked the normality assumptions by conducting multivariate normality tests (Shapiro, Mardia, Henze-Zirkler and Royston tests in R) and univariate normality tests (ShapiroWilk test in R, in combination with q-q plots and histograms) [1]. When the assumption of multivariate normality was not met, we executed transformations of the dependent variable. When transformation did not lead to multivariate normality, we used robust maximum likelihood estimation (MLR) to fit the model. P-values smaller than 0.05 were considered statistically significant. Data were analyzed according to the intention-to-treat (ITT) principle, as mentioned in the study protocol [2]. Missing data were analysed for random occurrence with Little’s MCAR test for missing data [3]. Multiple imputation was used for all variables to handle the missing data on item level when data was missing completely at random [4,5]. We handled missing data due to attrition using the full information maximum likelihood procedures incorporated in the growth model function of the Lavaan R package. Differences in number of dropouts between both groups were compared with a cross table and chi-square test.

*RQ1: Long-term effectiveness of online self-help training in reducing FCR and PD*

To answer the first research question (i.e., do the intervention and CAU group differ in the change in FCR/PD over time), a multigroup second order latent growth model was estimated. A second-order growth model estimates individual growth curves for each participant based on the individual FCR or PD item scores. A multigroup extension allows testing differences between interventions in the growth model parameters. We estimated the model separately for FCR and PD, because the multivariate model for both FCR and PD did not converge (most likely, the model was too large for the data). Also, since R package lavaan gave a warning for all models using robust maximum likelihood estimation (MLR) (i.e., “The variance-covariance matrix of the estimated parameters (vcov) does not appear to be positive definite! The smallest eigenvalue (= -7.784349e-16) is smaller than zero. This may be a symptom that the model is not identified”), we used maximum likelihood estimation (ML) to fit the model. Growth model analyses were performed with R statistical software (version 3.5.3), with software packages lavaan (R package for Structural Equation Modeling, version 0.6-9) and BaylorEdPsych (R-package used for checking missing data for random occurrences, version 0.5) [3,6–8]. The code used for the analyses in this study has been made openly available in the Open Science Framework (OSF) repository (<https://osf.io/2sg6m/>). Each of the FCR/PD models was built in five steps: Model 0 was the baseline model with five correlated longitudinal latent factors (one at each time-point), fitted separately for each treatment group. All correlations between the same item at different time points were freely estimated. Growth models assume longitudinal measurement invariance, implying that the measurement model of each outcome (FCR/PD) is invariant across time. To test this assumption, the loadings of the five longitudinal factors were constrained to be equal across time in Model 1, and the intercepts of individual items were constrained to be equal across time in Model 2. In Model 3, we added the latent growth factors (intercept and slope) to explain individual change in the outcome over time for each treatment group separately. Lastly, in Model 4 we tested the first research question by constraining the latent slopes of the intervention and CAU group to be equal.

Goodness-of-fit of the latent growth models was assessed with the maximum-likelihood chi-square statistic (χ^2^), Akaike Information Criterion (AIC), Bayesian Information Criterion (BIC), the root mean squared error of approximation (RMSEA), the comparative fit index (CFI), and the Tucker-Lewis index (TLI) [9–11]. The goodness of fit criteria for the fit indices are: CFI and TLI ≥ .95 and RMSEA ≤ .06 [10,11] . We used the χ^2^, AIC and BIC criteria to compare models, with higher χ^2^, and lower values of AIC and BIC, indicating a better model [10,11]. Moreover, we compared each set of nested models using chi-square difference test. When models with one or more parameter constraints did not show significantly worse fit in terms of chi-square, longitudinal measurement invariance can be concluded with respect to the constrained parameter type.

*RQ2: Relation between FCR and PD over time*

Answering the second research question (i.e., does the association between FCR and PD decrease over time), we used a multivariate multigroup growth model including both FCR and PD scores over time. However, as this multivariate second order growth model did not converge, we used a first order growth model that models the growth parameters based on FCR and PD factor scores at each time point instead of on the questionnaire item scores. We first estimated the factor scores of the FCR and PD measurement models at each of the five time points, and subsequently used these factor scores in a multivariate multigroup first order growth model (Model 5) to investigate how change in FCR scores over time was related to change in PD scores over time and how these relations differed between the CAU and intervention group. To test whether the correlations between FCR and PD were equal over time, we added restrictions in Models 6 and 7 respectively to the intervention and CAU group. We assessed and interpreted the goodness-of-fit of the latent growth models as described above.

*RQ3: Change trajectories in FCR over time and their predictors*

For the last research question (i.e., what are the characteristics of patients for whom the intervention worked), we modeled a repeated measures latent class analysis (RMLCA) within the intervention group. RMLCA analyses were performed with Latent Gold (version 5.0.0) [12]. The first step of this analysis was to identify the optimal number of latent classes where each class represents a different pattern of change in FCR over time. We used the Bayesian Information Criterion (BIC; tends to underestimate with low separation and in small sample sizes), Akaike Information Criterion (AIC; tends to overestimate with higher separation and in large sample sizes), and AIC3 (offers a good compromise between BIC and AIC) to determine the number of latent classes that best fitted the data [13,14]. Lower values of BIC, AIC and AIC3 indicate a better fit of a model relative to models with higher values [10,11]. When not all indices indicated the same number of latent classes, the model supported by most indices was chosen. The second step of the RMCLA was to investigate which predictor variables (as mentioned in the introduction) predict class membership, using Latent Gold's omnibus Wald test of differences between the latent classes on the predictor variables. Since we assessed multiple predictors, we adjusted the significance level using the Bonferroni-Holm correction [15]. For significant predictors, we used Z values >2 or <-2 to determine which class(es) significantly contributed to the predictor effects. Positive/negative values show that participants in that class show higher/lower than average scores on that predictor.

**References**

1. Korkmaz S, Goksuluk D, Zararsiz G: MVN: An R package for assessing multivariate normality. R J. 2014; 6:151–162.

2. van Helmondt SJ, van der Lee ML, de Vries J: Study protocol of the CAREST-trial: a randomised controlled trial on the (cost-) effectiveness of a CBT-based online self-help training for fear of cancer recurrence in women with curatively treated breast cancer. BMC Cancer. 2016; 16:527.

3. Beaujean AA: BaylorEdPsych: R Package for Baylor University Educational Psychology Quantitative Courses. 2012; .

4. Little RJA: A test of missing completely at random for multivariate data with missing values. J Am Stat Assoc. 1988; 83:1198–1202.

5. Resseguier N, Giorgi R, Paoletti X: Sensitivity anaylsis when data are missing not-at-random. Epidemiology. 2011; 22:282.

6. IBM Corp.: IBM SPSS Statistics for Windows, Version 23.0. 2014; .

7. R Core Team: R: A language and environment for statistical computing. 2017; .

8. Rosseel Y: lavaan: An R Package for Structural Equation Modeling. J Stat Softw. 2012; 48:1–36.

9. Curran PJ, Obeidat K, Losardo D: Twelve frequently asked questions about growth curve modeling. J Cogn Dev. 2010; 11:121–136.

10. Schreiber JB, Nora A, Stage FK, Barlow E a, King J: Reporting Modeling Analysis and Confirmatory Results : Equation Factor Review. J Educ Res. 2006; 99:323–337.

11. Hooper D, Coughlan J, Mullen MR: Structural equation modelling: Guidelines for determining model fit. Electron J Bus Res Methods. 2008; 6:53–60.

12. Vermunt JK, Magidson J: Technical guide for Latent GOLD 5.0: Basic, advanced, and syntax. Belmont, MA: Statistical Innovations Inc., 2013.

13. Batagelj V, Bock H-H, Ferligoj A, Žiberna A (eds): Data Science and Classification, Studies in classification, data analysis, and knowledge organization. Berlin: Springer-Verlag, 2006.

14. Lukočiene O, Vermunt JK: Determining the number of components in mixture models for hierarchical data. In: Studies in Classification, Data Analysis, and Knowledge Organization. Berlin/Heidelberg, Germany: Springer, 2010. p. 241–249.

15. Holm S: A simple sequentially rejective multiple test procedure. Scand J Stat. 1979; 65–70.
